# Artificial intelligence in oncology: promise, peril, and the future of patient–physician interaction

**DOI:** 10.3389/fdgth.2025.1633577

**Published:** 2025-11-04

**Authors:** Birpartap S. Thind, Che-Kai Tsao

**Affiliations:** ^1^ School of Medicine, California University of Science and Medicine, Colton, CA, United States; ^2^ Northwell Cancer Institute, New Hyde Park, NY, United States

**Keywords:** artificial intelligence, oncology, patient–physician interaction, chatbots, extended reality, communication, shared decision-making

## Abstract

Artificial intelligence (AI) is increasingly embedded in oncology. While initial technical evaluations emphasize diagnostic accuracy and efficiency, the impact on patient–physician interaction (PPI)—the foundation of trust, communication, comprehension, and shared decision-making—remains underexplored. In this review, we studied the current development of AI technology facing both physicians and patients with a focus in cancer care. Among different AI technologies, chatbots, large language model agents, and extended reality applications have shown the promise to date. Survey data suggest oncologists recognize AI's potential to augment efficiency but remain cautious about liability and the erosion of relational care. Key to future AI success in improving cancer care critically depends on design, validation, governance, and human guidance and gatekeeping in care delivery.

## 1 Introduction

Artificial Intelligence (AI) has started to enter nearly every facet of cancer care, including screening, diagnostics, treatment selection, and survivorship. As deep learning technology continues to improve, AI increasingly mediates how patients and clinicians communicate, decide, and make decisions. Patient-facing chatbots and LLMs can answer questions on demand, synthesize complex information into plain language, and scaffold decision discussions. Furthermore, immersive XR platforms can extend communication beyond text into embodied, visual explanations that may reduce anxiety and foster shared decision-making (SDM) for cancer patients. However, without appropriate guidance, this can result in, overstated confidence and miss nuance—undercutting trust at the bedside (1–6).

Professional discourse mirrors this ambivalence. Recent surveys show oncologists value AI for automating administrative tasks and enhancing efficiency, but express concern about accountability in error cases, unequal patient access, and loss of empathy in encounters. These attitudes highlight AI's double-edged role: a potential partner in communication, yet a possible culprit for disruption of trust if poorly governed.

This review situates oncology AI literature explicitly within the PPI lens. Unlike prior reviews focused on diagnostic accuracy, we examine how AI affects communication quality, comprehension, decisional conflict, satisfaction, and trust, while acknowledging professional perspectives and ongoing debates about responsibility.

## 2 Methods

We followed PRISMA 2020 guidance in a streamlined format appropriate for this review. Searches covered PubMed and Embase (no lower date limit; last update September 2025) using: (chatbot) AND (“artificial intelligence” OR AI) AND (cancer OR oncology) AND education. Filters included English, peer-reviewed, full-text. Exclusions: conference abstracts, books, letters, and editorials. Title/abstract screening was followed by full-text assessment against prespecified inclusion criteria: oncology context; chatbot/LLM or closely related patient-facing AI; and PPI-relevant outcomes (e.g., communication quality, comprehension/readability, decisional conflict, trust, satisfaction, equity).

**Figure 1:**
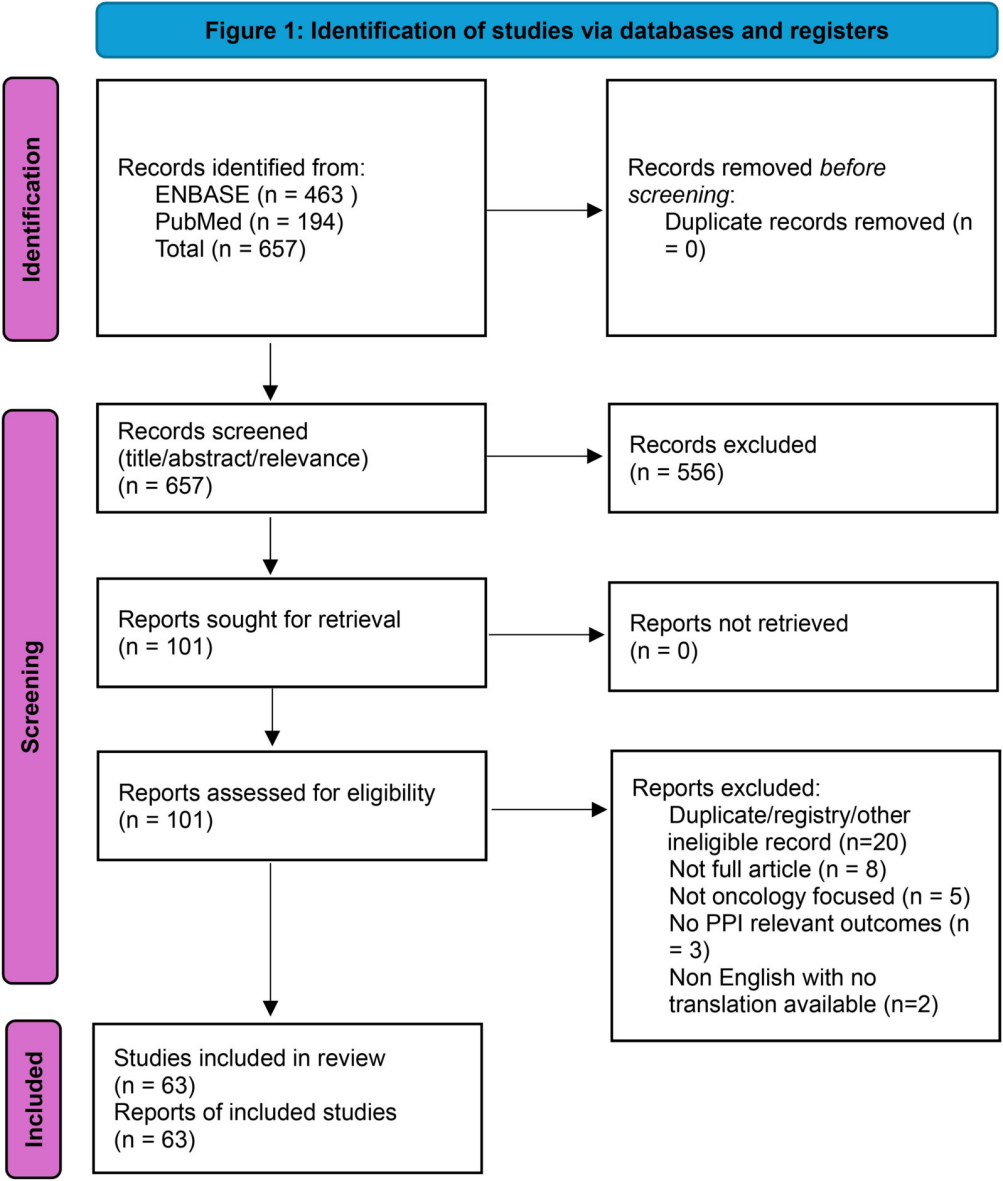
Identification of studies via databases and registers.

We identified 657 records (PubMed *n* = 194, Embase *n* = 463). After screening titles/abstracts, 101 reports were retrieved for full-text review. Following eligibility assessment, 63 unique studies met inclusion criteria. Several duplicate entries were identified across databases (e.g., trial registrations and overlapping records) and removed during full-text deduplication. Two XR breast cancer studies suggested by peer reviewers were screened against the same criteria and included. Data extraction captured cancer type, AI modality, care phase, study design, comparators, PPI outcomes, and guardrail domains. Supplementary Table 1 lists all included studies. A PRISMA-style flow diagram is presented in Figure 1.

## 3 Results

### 3.1 Overview

A large subset of the included studies evaluated chatbots and LLMs answering cancer questions or assisting reasoning. Across studies, chatbots often produced readable, detailed answers but showed variable accuracy and inconsistent citation behavior, with risks of fabricated references (4–6). Multimodal systems did not consistently outperform text-only models and struggled on free-text reasoning (3). In imaging, randomized evidence from mammography (MASAI trial) shows AI can reduce reading workload while maintaining safety—indirectly supporting PPI by freeing clinician time (7). XR studies in breast cancer suggest immersive education lowers anxiety and strengthens SDM (5, 6).

### 3.2 Chatbots and conversational agents

A 2023 JAMA Oncology analysis found chatbots produced high-quality consumer information using validated instruments, though readability was at a college level and actionability limited (1). In 2024, JAMA Oncology reported that chatbots generated longer, more detailed responses to patient questions than oncologists, with comparable empathy ratings but unresolved safety concerns (2). A 2024 JAMA Network Open study found multimodal chatbots were not consistently more accurate than unimodal ones, especially on open-ended tasks (3). Head-and-neck oncology work highlighted citation fabrication as a major limitation for trust (4). Key evaluations of oncology chatbots/LLMs are summarized in Table 1, including landmark studies as well as prostate theranostics, randomized patient-facing chatbot trials, and cancer genetics applications (8–10).

**Table 1:** AI chatbots/LLMs in oncology and PPI outcomes.

Ref#	Study (journal, year)	Cancer context	Modality	PPI outcomes	Key findings
1	JAMA Oncol 2023	Mixed cancers	Multiple chatbots	Info quality; readability	High quality, low actionability, college-level reading
2	JAMA Oncol 2024	Online patient Qs	ChatGPT, Claude	Quality; empathy	Longer, more detailed answers; empathy ratings high
3	JAMA Netw Open 2024	Oncology cases	Multimodal chatbots	Accuracy	Multimodal not consistently better; free-text weaker
4	Eur Arch ORL 2024	Head & Neck	ChatGPT 3.5/4	Citation reliability	Fabricated/inaccurate references
8	Front Oncol 2024	Prostate cancer	ChatGPT-4, Bard	Accuracy, readability	Accurate Q&A on theranostics; readability moderate
9	Eur Urol Open Sci 2024	Prostate cancer	Conversational agent	Comprehension; non-inferiority	RCT: chatbot not inferior to physicians for pre-consult education
10	JAMA Netw Open 2024	Cancer genetics	BRIDGE chatbot	Genetic counseling access	RCT: improved service delivery vs. standard referral

### 3.3 Decision-support and digital pathology tools

Tools such as OncoKB and AI-derived pathology biomarkers translate genomics and histology into treatment recommendations. For example, OncoKB categorizes cancer mutations by therapeutic actionability, which helps oncologists frame genomic results for patients during molecular tumor board discussions. Similarly, Armstrong et al. demonstrated that an AI-derived pathology biomarker could predict prostate cancer patients' benefit from androgen deprivation therapy, guiding physician–patient discussions on intensifying treatment. These tools influenced PPI indirectly by shaping clinical dialogue, particularly when clinicians contextualized outputs for patients. Survey studies reinforce that clinicians expect to remain accountable for interpretation, underscoring the need for AI as support rather than surrogate (11, 12).

### 3.4 XR/metaverse applications

Two breast cancer studies illustrated immersive AI tools' ability to clarify surgical planning and survivorship education. For instance, one study used VR to simulate breast reconstruction outcomes, enabling patients to better visualize surgical choices and report less decisional conflict. Another integrated XR into survivorship education, where patients demonstrated higher comprehension of follow-up care and greater satisfaction with counseling. Patients exposed to XR/VR environments overall reported improved comprehension, reduced anxiety, and greater engagement in SDM. These are summarized in Table 2.

**Table 2:** Xr/metaverse applications.

Ref#	Study	Cancer context	Modality	PPI outcomes	Key findings
5	J Clin Med 2024	Breast cancer	XR/Metaverse	Education; anxiety; SDM	Improved comprehension, reduced anxiety
6	J Clin Med 2024	Breast surgery	3D + VR/AR	Engagement; education	Enhanced understanding of surgical options

### 3.5 Risks and guardrails

As advancement in AI application has entered oncology care, several potential risks have been identified: (1) unverifiable claims undermining trust (1–4); (2) bias and inequity in access and training data; (3) workflow misfit and unclear liability raising clinician burden; and (4) relational harms, such as reduced empathy when technology displaces human dialogue. Guardrails include verifiability, transparency, clinician oversight, equity-focused design, and reporting standards such as CONSORT-AI, TRIPOD + AI, and CHART (13–15).

### 3.6 Professional perspectives

Surveys highlight that oncologists value efficiency gains but remain cautious about liability, accountability, and loss of empathy. For example, one international survey found that while 75% of oncologists believed AI could streamline documentation, fewer than 30% trusted AI with independent decision-making. Another U.S.-based survey reported that most respondents expected empathy and accountability to remain clinician responsibilities, even if AI were adopted (16–20). Table 3 summarizes key surveys and professional perspectives, including attitudes toward accountability, willingness to adopt AI, and perceptions of patient trust.

**Table 3:** Surveys and professional perspectives on AI in oncology.

Ref#	Study (journal, year)	Population	Key findings
16	JAMA Netw Open 2024	Oncologists	Efficiency gains; liability concerns
17	JCO Clin Cancer Inform 2023	Oncologists	Accountability, adoption attitudes
18	Cancer 2024	Oncologists, patients & family members	Opportunities and challenges
19	Eur Urol Focus 2024	Patients with prostate cancer	Trust in AI-based decision-making
20	J Med Internet Res 2024	Providers & patients	Benefits and limitations of chatbots

## 4 Discussion

AI in oncology holds potential to reshape the contours of patient–physician communication. At the most basic level, chatbots make cancer knowledge more accessible, translating medical jargon into plain language (1–3). Several studies highlight that patients value such accessibility, particularly in low-resource settings where direct oncologist consultation may be limited. But accessibility without accuracy is hollow; hallucinations and citation fabrication remain major threats (4). Patients increasingly verify AI outputs online, and fabricated references can erode trust more rapidly than incomplete answers. Trust, once broken, is difficult to restore—making verifiability a non-negotiable design requirement. In practice, this points to a model where AI outputs are mediated through human gatekeeping mechanisms: clinicians or trained staff vet AI-generated information before it reaches patients, or present AI summaries with their own interpretive framing. Such oversight maintains the benefits of accessibility while safeguarding against misinformation, aligning with survey data showing that patients and clinicians alike expect physicians to remain accountable for final interpretation.

A second central theme is the evolving balance of empathy across oncology settings. Evidence from 2024 suggests some chatbots generated responses rated as more empathetic than oncologists, raising the possibility that tone-optimized AI could complement strained clinician bandwidth in high-volume settings (2). Beyond general cancer information, chatbots are also being deployed in pediatric, survivorship, and palliative care contexts, where communication needs are unique. For instance, co-design pilots in pediatric and AYA oncology demonstrated feasibility and empowerment but also highlighted literacy barriers (21). Survivorship interventions reported improved engagement and self-management, though accuracy limitations persisted, and palliative care evaluations found chatbots useful in structuring sensitive conversations but constrained in empathy and nuance (22). Professional surveys reinforce this nuance, noting that oncologists value efficiency gains but remain cautious about accountability and relational harms (16–20). Taken together, these findings suggest that chatbots may best serve as supplemental tools—able to introduce information and reinforce self-management—while clinicians remain central for contextual, emotional, and relational care.

XR/Metaverse studies expand the conversation. By immersing patients in their own anatomy and surgical options, XR fosters active participation in decision-making (5, 6). These immersive approaches may be especially impactful in breast cancer surgery, where anxiety and decisional conflict are common. The XR evidence suggests AI can scaffold communication not only through words but also through visualization. Importantly, these approaches can be positioned to complement rather than compete with text-based chatbots or LLMs: while conversational agents provide accessible, on-demand explanations, XR models offer experiential visualization that can reinforce those explanations and anchor them in the patient's own body. Prototype “virtual nursing” models already blend conversational AI with immersive guidance, hinting at a future where multimodal platforms combine verbal and visual support to enhance comprehension and engagement. The question is whether such benefits will generalize beyond single-institution studies in high-resource settings.

Decision-support systems (e.g., OncoKB, AI pathology biomarkers) occupy another layer. Although not directly conversational, they shape PPI indirectly: the quality of oncologist–patient discussions hinges on how confidently and transparently clinicians can explain AI-derived recommendations (11, 12). For example, the FDA-cleared ArteraAI digital pathology test predicts which prostate cancer patients may benefit from androgen deprivation therapy, enabling more precise treatment discussions. Such tools illustrate how AI-derived recommendations can enhance precision medicine while placing responsibility on clinicians to interpret results responsibly. Here, liability concerns loom large. Surveys show most oncologists expect to remain responsible for AI-informed decisions, highlighting the need for governance frameworks that preserve accountability while distributing responsibility fairly.

Across all modalities, the equity dimension cannot be ignored. Bias in training data disproportionately affects marginalized populations. Readability levels skew above average literacy. And XR platforms risk widening the digital divide. Addressing these inequities requires deliberate design choices—multilingual support, culturally competent training data, and evaluation in diverse populations (16–20). Equity is not a side issue but a central determinant of whether AI improves or undermines relational care. Sustained investment is also needed: technologies must be developed with inclusive language models, low-bandwidth XR options, and educational scaffolds that adapt to varied health literacy. Such investments can help ensure that AI reduces rather than reinforces disparities.

Our synthesis aligns with and extends professional discourse. ASCO and other professional bodies stress the ethical stakes of AI in oncology, calling for transparency, interpretability, and explicit measurement of communication outcomes (23). Yet most published studies stop short at technical accuracy or single-use case evaluations. Few directly measure trust, decisional conflict, or patient satisfaction, leaving a gaping evidence gap. Looking ahead, several priorities emerge. First, prospective trials must move beyond accuracy and efficiency to relational endpoints such as trust, comprehension, decisional conflict, and satisfaction, using validated tools. Without such measures, we cannot know whether AI is truly strengthening PPI. Second, hybrid human–AI models deserve systematic evaluation: chatbots may triage questions, XR can visualize options, and decision-support systems can suggest treatments, but oncologists must integrate, contextualize, and humanize these outputs. Third, governance and accountability frameworks are essential. Surveys show that oncologists demand clear lines of responsibility, without which adoption will remain limited and trust fragile (16–18). Fourth, equity and access must be central: tools must be evaluated across literacy levels, languages, and socioeconomic contexts, and XR interventions must be assessed for accessibility to avoid deepening disparities (16–20). Finally, education and training are needed to prepare the next generation of oncologists to critically appraise AI outputs, integrate them into relational communication, and maintain empathy.

## 5 Conclusion

Artificial intelligence in oncology, as it currently stands, it is a double-edged sword whose relational impact is critically dependent on design, governance, and integration. Chatbots and XR show promise for education, engagement, and anxiety reduction, while decision-support tools can enhance the diagnostic and treatment precision leading to better quality of care. Yet, risks of hallucinations, inequity, liability confusion, and empathy erosion are substantial risks at hand. Oncology must commit to designing, evaluating, and governing AI in ways to supplement and partner with clinicians, and in doing so strengthen the human connection at the heart of care. Future studies should prioritize relational outcomes and partnership, ensuring that AI serves as an ally in patient–physician interaction rather than an interloper.
