# Supplementary material for: Artificial intelligence in oncology: promise, peril, and the future of patient–physician interaction
Source: Front Digit Health. 2025 Nov 4;7:1633577. doi: 10.3389/fdgth.2025.1633577 (PMC12623327; doi:10.3389/fdgth.2025.1633577)
Supplement: Supplementary file 1 [file Table1.pdf]

Supplementary Material

Year	Title	Journal	DOI
2025	A Comparison of Prostate Cancer Screening Information Quality on Standard and Advanced Versions of ChatGPT, Google Gemini, and Microsoft Copilot: A Cross-Sectional Study	Am J Health Promot	10.1177/08901171251316371
2025	A literature review on the role of artificial intelligence–based chatbots in patient education in colorectal surgery	Surgery	10.1016/j.surg.2025.109393
2025	A Study on Breast Cancer Patient Care Using Chatbot and Video Education for Radiation Therapy: A Randomized Controlled Trial	Int J Radiat Oncol Biol Phys	10.1016/j.ijrobp.2024.12.012
2025	Accuracy, readability, and understandability of large language models for prostate cancer information to the public	Prostate Cancer Prostatic Dis.	10.1038/s41391-024-00826-y
2025	An Assessment of ChatGPT's Responses to Common Patient Questions About Lung Cancer Surgery: A Preliminary Clinical Evaluation of Accuracy and Relevance	J. Clin. Med.	10.3390/jcm14051676
2025	Application of AI Chatbot in Responding to Asynchronous Text-Based Messages From Patients With Cancer: Comparative Study	J Med Internet Res	10.2196/67462
2025	Are AI chatbots concordant with evidence-based cancer screening recommendations?	Patient Educ. Couns.	10.1016/j.pec.2025.108677
2025	Assessing the Quality and Reliability of ChatGPT's Responses to Radiotherapy-Related Patient Queries: Comparative Study With GPT-3.5 and GPT-4	JMIR Cancer	10.2196/63677

2025	Benchmarking LLM chatbots' oncological knowledge with the Turkish Society of Medical Oncology's annual board examination questions	BMC Cancer	10.1186/s12885-025-13596-0
2025	Chatbots for breast cancer education: a systematic review and meta-analysis	Supportive Care Cancer	10.1007/s00520-024-09096-9
2025	ChatGPT as a patient education tool in colorectal cancer—An in-depth assessment of efficacy, quality and readability	Colorectal Dis.	10.1111/codi.17267
2025	Design and Validation of a Chatbot-Based Cervical Cancer Screening Decision Aid for Women Experiencing Socioeconomic Disadvantage: User-Centered Approach Study	JMIR Cancer	10.2196/70251
2025	Empowering breast cancer clients through AI chatbots: transforming knowledge and attitudes for enhanced nursing care	BMC Nurs	10.1186/s12912-025-03585-w
2025	Evaluating Artificial Intelligence Chatbots in Oral and Maxillofacial Surgery Board Exams: Performance and Potential	J. Oral Maxillofac. Surg.	10.1016/j.joms.2024.11.007
2025	Evaluation of AI-Based Chatbots in Liver Cancer Information Dissemination: A Comparative Analysis of GPT, DeepSeek, Copilot, and Gemini	Oncology	10.1159/000546726
2025	Evidence-Based Analysis of AI Chatbots in Oncology Patient Education: Implications for Trust, Perceived Realness, and Misinformation Management	J Cancer Educ	10.1007/s13187-025-02592-4
2025	Large Language Models in Healthcare: A Bibliometric Analysis and Examination of Research Trends	J. Multidiscip.Healthc.	10.2147/JMDH.S502351

2025	Leveraging artificial intelligence-mediated communication for cancer prevention and control and drug addiction: A systematic review	Transl. Behav. Med.	10.1093/tbm/ibaf007
2025	Perceptions and Attitudes of Chinese Oncologists Toward Endorsing AI-Driven Chatbots for Health Information Seeking Among Patients with Cancer: Phenomenological Qualitative Study	J Med Internet Res	10.2196/71418
2025	Protocol for an umbrella review of systematic reviews evaluating the efficacy of digital health solutions in supporting adult cancer survivorship care	PLoS ONE	10.1371/journal.pone.0322100
2025	The Effectiveness and Feasibility of Conversational Agents in Supporting Care for Patients With Cancer: Systematic Review and Meta-Analysis	J. Med. Internet Res.	10.2196/76968
2025	The Extent to Which Artificial Intelligence Can Help Fulfill Metastatic Breast Cancer Patient Healthcare Needs: A Mixed-Methods Study	Curr. Oncol.	10.3390/curroncol32030145
2025	The role of natural language processing in improving cancer care: A scoping review with narrative synthesis	Artif. Intell. Med.	10.1016/j.artmed.2025.103227
2025	The Usability and Experience of Artificial Intelligence-Based Conversational Agents in Health Education for Cancer Patients: A Scoping Review	J Clin Nurs	10.1111/jocn.70020
2025	Theory-based chatbot for promoting colorectal cancer screening in a community setting in Hong Kong: study protocol of a randomised controlled trial	BMJ Open	10.1136/bmjopen-2025-103857

2025	User testing of a previsit chatbot developed for population genomic screening and counseling	J Genet Couns	10.1002/jgc4.70090
2025	Using a Multilingual AI Care Agent to Reduce Disparities in Colorectal Cancer Screening for Higher Fecal Immunochemical Test Adoption Among Spanish-Speaking Patients: Retrospective Analysis	J. Med. Internet Res.	10.2196/71211
2025	Utility of Chatbot Literature Search in Radiation Oncology	J Cancer Educ	10.1007/s13187-024-02547-1
2024	Accuracy of an Artificial Intelligence Chatbot's Interpretation of Clinical Ophthalmic Images	JAMA Ophthalmol.	10.1001/jamaophthalmol.2024.0017
2024	Assessing the response quality and readability of chatbots in cardiovascular health, oncology, and psoriasis: A comparative study	Int. J. Med. Informatics	10.1016/j.ijmedinf.2024.105562
2024	Can Artificial Intelligence “Hold” a Dermoscope?—The Evaluation of an Artificial Intelligence Chatbot to Translate the Dermoscopic Language	Diagn.	10.3390/diagnostics14111165
2024	Chatbots in Cancer Applications, Advantages and Disadvantages: All that Glitters Is Not Gold	J. Pers. Med.	10.3390/jpm14080877
2024	ChatGPT as an aid for pathological diagnosis of cancer	Pathol. Res. Pract.	10.1016/j.prp.2023.154989
2024	Communicative competence of generative artificial intelligence in responding to patient queries about colorectal cancer surgery	Int. J. Colorectal Dis.	10.1007/s00384-024-04670-3

2024	Effectiveness of the Medical Chatbot PROSCA to Inform Patients About Prostate Cancer: Results of a Randomized Controlled Trial	Eur Urol Open Sci	10.1016/j.euros.2024.08.022
2024	Empowering patients: how accurate and readable are large language models in renal cancer education	Front. Oncol.	10.3389/fonc.2024.1457516
2024	Enhancing Readability of Online Patient-Facing Content: The Role of AI Chatbots in Improving Cancer Information Accessibility	JNCCN J. Nat. Compr. Cancer Netw.	10.6004/jnccn.2023.7334
2024	Evaluating the Accuracy of ChatGPT in Common Patient Questions Regarding HPV+ Oropharyngeal Carcinoma	Ann. Otol. Rhinol. Laryngol.	10.1177/00034894241259137
2024	Evaluating the Efficacy of ChatGPT as a Patient Education Tool in Prostate Cancer: Multimetric Assessment	J. Med. Internet Res.	10.2196/55939
2024	Evaluation of Oropharyngeal Cancer Information from Revolutionary Artificial Intelligence Chatbot	Laryngoscope	10.1002/lary.31191
2024	Exploring the Role of Artificial Intelligence Chatbots in Preoperative Counseling for Head and Neck Cancer Surgery	Laryngoscope	10.1002/lary.31243
2024	How Well Do Artificial Intelligence Chatbots Respond to the Top Search Queries About Urological Malignancies?	Eur. Urol.	10.1016/j.eururo.2023.07.004
2024	Innovations in Medicine: Exploring ChatGPT's Impact on Rare Disorder Management	Genes	10.3390/genes15040421
2024	Integrating artificial intelligence in renal cell carcinoma: evaluating ChatGPT's performance in educating patients and trainees	Transl. Cancer Res.	10.21037/tcr-23-2234

2024	Performance of large language models (LLMs) in providing prostate cancer information	BMC Urol.	10.1186/s12894-024-01570-0
2024	Physician and Artificial Intelligence Chatbot Responses to Cancer Questions From Social Media	JAMA Oncol	10.1001/jamaoncol.2024.0836
2024	Recommendation for gastroenterologists: Increase in the identification rate of hereditary gastrointestinal tumor predisposition syndromes?	Coloproctology	10.1007/s00053-024-00775-z
2024	Reliability of artificial intelligence chatbot responses to frequently asked questions in breast surgical oncology	J Surg Oncol	10.1002/jso.27715
2024	Roles, Users, Benefits, and Limitations of Chatbots in Health Care: Rapid Review	J. Med. Internet Res.	10.2196/56930
2024	The doc versus the bot: A pilot study to assess the quality and accuracy of physician and chatbot responses to clinical questions in gynecologic oncology	Gynecol Oncol Rep	10.1016/j.gore.2024.101477
2024	The Use of Chatbots in Head and Neck Mucosal Malignancy Treatment Recommendations	Otolaryngol. Head Neck Surg.	10.1002/ohn.818
2023	A wearable chatbot-based model for monitoring colorectal cancer patients in the active phase of treatment	Healthc. Anal.	10.1016/j.health.2023.100257
2023	An artificial intelligence-based chatbot for prostate cancer education: Design and patient evaluation study	Digit Health	10.1177/20552076231173304
2023	Assessment of Artificial Intelligence Chatbot Responses to Top Searched Queries about Cancer	JAMA Oncol.	10.1001/jamaoncol.2023.2947

2023	The Use of Chatbots in Oncological Care: A Narrative Review	Int J Gen Med	10.2147/IJGM.S408208
2022	Ask Rosa – The making of a digital genetic conversation tool, a chatbot, about hereditary breast and ovarian cancer	Patient Educ. Couns.	10.1016/j.pec.2021.09.027
2022	Digital technologies in cancer care: a review from the clinician's perspective	J. Comp. Eff. Res.	10.2217/cer-2021-0263
2022	The Use of Chatbots as Supportive Agents for People Seeking Help with Substance Use Disorder: A Systematic Review	Eur. Addict. Res.	10.1159/000525959
2021	Chatbot for Health Care and Oncology Applications Using Artificial Intelligence and Machine Learning: Systematic Review	JMIR Cancer	10.2196/27850
2021	Development and early feasibility of chatbots for educating patients with lung cancer and their caregivers in Japan: Mixed methods study	JMIR Cancer	10.2196/26911
2021	Using chatbots to screen for heritable cancer syndromes in patients undergoing routine colonoscopy	J Med Genet	10.1136/jmedgenet-2020-107294
2019	Healthcare ex Machina: Are conversational agents ready for prime time in oncology?	Clin. Transl. Radiat. Oncol.	10.1016/j.ctro.2019.04.002
2018	Clinical integration of digital solutions in health care: An overview of the current landscape of digital technologies in cancer care	JCO Clin. Cancer Inform.	10.1200/CCI.17.00159
